# AKT-mediated phosphorylation of TWIST1 is essential for breast cancer cell metastasis

**DOI:** 10.3906/biy-1912-74

**Published:** 2020-08-19

**Authors:** Mustafa Gökhan ERTOSUN, Suray PEHLİVANOĞLU, Sayra DİLMAÇ, Gamze TANRIÖVER, Osman Nidai ÖZEŞ

**Affiliations:** 1 Department Plastic, Reconstructive and Aesthetic Surgery, Faculty of Medicine, Akdeniz University, Antalya Turkey; 2 Department of Molecular Biology, Faculty of Science, Necmettin Erbakan University, Konya Turkey; 3 Department of Histology and Embryology, Faculty of Medicine, Akdeniz University, Antalya Turkey; 4 ALTAY Therapeutics, San Francisco, California USA

**Keywords:** TWIST1, breast cancer, metastasis

## Abstract

Previously, it was shown that human TWIST1 (basic helix-loop-helix (b-HLH) is phosphorylated by Akt kinase at S42, T121, and S123. To show in vivo effect of these phosphorylations, we created mouse TWIST1 expression vector and converted the codons of S42, T125, and S127 to unphosphorylatable alanine and phosphorylation mimicking Glutamic acid. We hypothesized that alanine mutants would inhibit the metastatic ability of 4T1 cells while glutamic acid mutants would convert nonmetastatic 67NR cells into metastatic phenotype. To confirm this hypothesis, we created metastatic 4T1 and nonmetastatic 67NR cells expressing alanine mutants and glutamic acid mutants mouse TWIST1, respectively. Then, we injected 1 × 10^6^ 67NR and 1 × 10^5^ 4T1 cells overexpressing mutants of TWIST1 into the breast tissue of BALB/c mice. At the end of the 4th week, we sacrificed the animals, determined the numbers of tumors at lungs and liver. Although 67NR cells overexpressing wild-type TWIST1 did not show any metastasis, cells overexpressing S42E and T125E mutants showed 15–30 macroscopic metastasis to liver and lungs. Parallel to this, 4T1 cells expressing S42A and T125A mutants of TWIST1 showed no macroscopic metastasis. Our results indicate that phosphorylation of S42 and T125 by AKT is essential for TWIST1-mediated tumor growth and metastasis.

## 1. Introduction

TWIST1 has been first characterized by its association with the embryonic development of Saethre–Chotzen syndrome and these individuals possess single functional copy (haploinsufficiency) of TWIST1, and this haploinsufficiency causes incomplete ossification and skull development (Chen and Behringer, 1995; Howard et al., 1997; el Ghouzzi et al., 1997; Qin et al., 2012). Studies related to this have shown that TWIST1 inhibits ossification by inhibiting the activities of RUNX2 and MyoD1, which are responsible for osteogenic and myogenic differentiation, respectively (Rice et al., 2000; Bialek et al., 2004; Connerney et al., 2006). Recent studies have shown that TWIST1 plays significant role in tumorigenesis and in line with this, TWIST1 inhibits apoptosis (Connerney et al., 2006), induces the expression of oncogenes such as STAT3 (Yousfi et al., 2002), AKT2 (Gong and Li, 2002), p14ARF (Funato et al., 2001), and accelerates p53 degradation (Xu et al., 2013). Parallel to all these, TWIST1 expression is upregulated in many types of cancer including glioma (Xu et al., 2017), stomach (Mironchik et al., 2005), liver (Cheng et al., 2007), endometrium (Cheng et al., 2008), breast (Xue et al., 2012), prostate cancer (Elias et al., 2005), angiogenesis (Yan-Qi et al., 2007), and epithelial-mesenchymal transition (Lee et al., 2006). TWIST1 induces the expression of N-cadherin while suppressing the expression of the epithelial marker, E-cadherin (Lee et al., 2006; Kyo et al., 2006). As we were conducting our studies to show the possible mechanism of TWIST1 activation by AKT, Xue et al. (2012) showed that TWIST1 is phosphorylated by AKT, and this phosphorylation mediates cross-talk between PI3K/AKT and TGF-B-signaling axes (Alexander et al., 2006). 

TWIST1 protein has three AKT phosphorylation sites in the consensus phosphorylation motif, RXRXXS/T. These sequences in human are RKRRSS42, RERQRT121, RQRTQS123, and in mouse are RKRRSS42, RERQRT125, and RQRTQS127. From that time, AKT-mediated phosphorylation of TWIST1 is already shown (Alexander et al., 2006), we wanted to investigate the effect of our mutants on growth, metastasis, and induction of TWIST1 induced Vimentin and N-cadherin in breast cancer cells 4T1 and 67NR cells as well. We found that nonmetastatic 67NR cells expressing glutamic acid mutants of TWIST1 can, in fact, metastasize to both liver and lungs. In contrast, metastatic 4T1 cells expressing alanine mutants of TWIST1 can no longer show any macroscopic metastasis to the liver or lungs. Also, while alanine mutants of TWIST1 diminish TWIST1-induced induction of N-cadherin and vimentin, glutamic acid mutants significantly induced expression of these genes in both cell types. Collectively, our results clearly show that AKT-mediated phosphorylation of TWIST1 is essential TWIST1-induced gene induction and cancer cell metastasis.

## 2. Materials and methods

###  2.1. Construction of TWIST1 expression vector. 

Mouse TWIST1 cDNA was cloned inframe into pcDNA3.1A as BamHI fragment by using forward and reverse primers shown below. Polymerase chain reaction (PCR) protocol was applied with Taq DNA polymerase (Fermentas). PCR: 1X reaction buffer, 2M MgCl_2_, 1 pmol of each primer, 2µ Tag DNA polymerase, 100 ng DNA, and 5% DMSO. PCR conditions were: denaturation at 94 oC for 5 min, 35 cycles of steps at 94 oC for 45 s, 58 oC for 45 s, 72 oC for 1 min, final extension was done at 72 oC for 7 min. The PCR products and the pcDNA3.1A were digested with BamHI and ligated with T4-DNA ligase at 16 oC for 1 h, then the ligation products were transformed into DH5a, plasmids were isolated from clones, and ligated the insert orientation was confirmed via ApaI restriction endonuclease digestion. Transfection of TWIST1 expression vector into 293 cells was performed using Ca-phosphate transfection protocol. Briefly, 30 ug of plasmid DNA was resuspended in 1 mL of 2X HEPES buffer (pH: 7.05) and 1 mL of 0.25M CaCL2 was added dropwise and the mixture was incubated at room temperature for 30 min, then CaCL_2_-DNA complex was added onto cells dropwise. The transfected cells were incubated for 72 h, the lysates were prepared for Western blotting.

Forward Pri.: 5’-cgcggatccgccaccatgatgcaggacgtgtccagc

tcgccagtctcgccgg-3’.

Reverse Pri: 5-cgcggatccgtgggacgcggacatggaccaggcccc-3,

Site Directed Mutagenesis: alanine (A) and glutamic acid (E) mutations of TWIST1 were generated by site directed mutagenesis (SDM) by using PFU DNA polymerase (Fermentas). Mutagenesis primers used for mouseTWIST1 are;

S42A; caagagacgcagcgcgcggcgcagcgcg, S42E; caagagacg

cagcgagcggcgcagcgcg, 

T125A; agcgccagcgcgcccagtcgctg, T125E; ggagcgccagcg

cgagcagtcgctgaac, 

S127A; cagcgcacgcaggcgctgaacgagg, S127E; ccagcgcacg

caggagctgaacgaggcg. 

The generation of mutants was confirmed by DNA sequencing using ABI-3130 (Figure S1).

### 2.2. Cell culture, cell lines, reagents, and antibodies

67NR and 4T1 cells were cultured in DMEM (Biochrom) medium supplemented with 10% FBS (Biochrom) and 1% (v/v) Pen-Strep Amp. solution (Biological Industries) in a humidified 5% CO_2_ incubator at 37 oC. For the generation of stable transfectants, cells were plated 24 h before transfection and transfected with 30 ug plasmid DNA using Ca-phosphate standard procedure. Stable clones were selected with 800 μg/mL of neomycin/G418 (Roche) for 8 weeks. Anti-Twist (sc-81417), anti-N-cadherin (sc-59987) antivimentin (V9) (sc-6260) were from Santa Cruz Biotechnology, Inc. (Santa Cruz, CA, USA). 

### 2.3. Western blot 

Protein concentrations of whole cell lysates obtained in lysis buffer (20 mM Tris-HCl (pH 7.4), 150 mM NaCl, 1.2% Triton X-100, 1 mM EGTA, 1 mM EDTA, 1 mM PMSF, 0.15 U/mL aprotinin, 10 g/mL leupeptin, 10 g/mL pepstatin A, and 1 mM sodium orthovanadate) were determined using the Bradford method. Equal amounts of protein were fractionated by SDS-PAGE on 10% polyacrylamide gels and transferred overnight to Immobilon-P PVDF membranes (Millipore, Bedford, MA, USA). The membrane was blocked with 1% BSA in PBS-T buffer for 2 h at room temperature and probed with anti-TWIST1 (Santa Cruz, Cat:#sc-81417) primary antibody for 1 h at room temperature. After washing, the membranes were incubated with peroxidase-conjugated secondary antibody (Biorad, Cat No:# 1706516) for 1 h, and proteins were detected using Clarity ECL western blotting substrate (Bio-Rad, 1705061).

### 2.4. In vivo breast cancer model 

Wild-type 4T1 and stable transfected 4T1 cells (10^5^ cells per mouse) were injected orthotopically into the right upper mammary gland of 8–10-week-old BALB/c mice. Identically wild-type 67NR and stable transfected 67NR cells (10^6^ cells per mouse) were injected into the mammary pad of each mouse. Primary tumor, lung, and liver tissues were removed 27 days after injection and fixed in 4% formalin and embedded in paraffin.

### 2.5. Metastasis assay

Five serial sections (5 μm thick) were prepared from each tumor sample. Slides were stained with hematoxylin and eosin to determine metastasis lesions. Twenty pictures were taken randomly with 20× magnification of each mouse for metastasis assay. Metastatic lesions were selected and measured as mm^2^ with Spot advanced 4.6 program (Diagnostic Instruments, Sterling Heights, MI).

### 2.6. Immunohistochemistry

Tissues were fixed in 4% formaldehyde (Merck; #1040032500, NJ, USA) solution for 24 h and embedded in paraffin. Sections of 5 μm were cut and collected on positive charged slides (Thermo Fisher; #9951plus, MA, USA) and incubated overnight at 56 °C. Tissues were deparaffinized in xylene (Merck; #1086852500, NJ, USA) and rehydrated with ethanol (Merck; #1009832500, NJ, USA). Sections were then treated with 10 mM citrate buffer, pH 6.0, for 5 min in a microwave oven. Following washes in phosphate buffered saline (PBS), sections were incubated in a universal blocking reagent (Scytek; #AAA125, UT, USA) for 7 min at room temperature. Subsequently, sections were incubated overnight at 4 °C with E-cadherin (1:50, Santa Cruz; #sc8426, TX, USA), N-Cadherin (1:50, Santa Cruz; #sc7939, TX, USA) and vimentin (1:50, Santa Cruz; #sc373717, TX, USA) antibodies. After several washes with PBS, sections were incubated with biotinylated goat antirabbit (1:400; Vector Lab., #BA-1000, CA, USA) and biotinylated horse antimouse (1:400; Vector Lab., #BA-2000, CA, USA) secondary antibodies for 1 h at room temperature and rinsed with PBS. Visualization was provided via streptavidin-peroxidase complex (Scytek; #SHP125, UT, USA) and diaminobenzidine (Sigma; #D4186-50, MO, USA). Sections were counterstained with Mayer’s hematoxylin (Merck; #1092490500, NJ, US) and mounted with Kaisers Glycerin Gelatine (Merck; #1079610100, NJ, US). Slides were examined using the Zeiss-Axioplan microscope (Oberkochen, Germany). 

## 3. Results

### 3.1. Cloning, mutagenesis, and expression of mouse TWIST1

**Figure 1:**
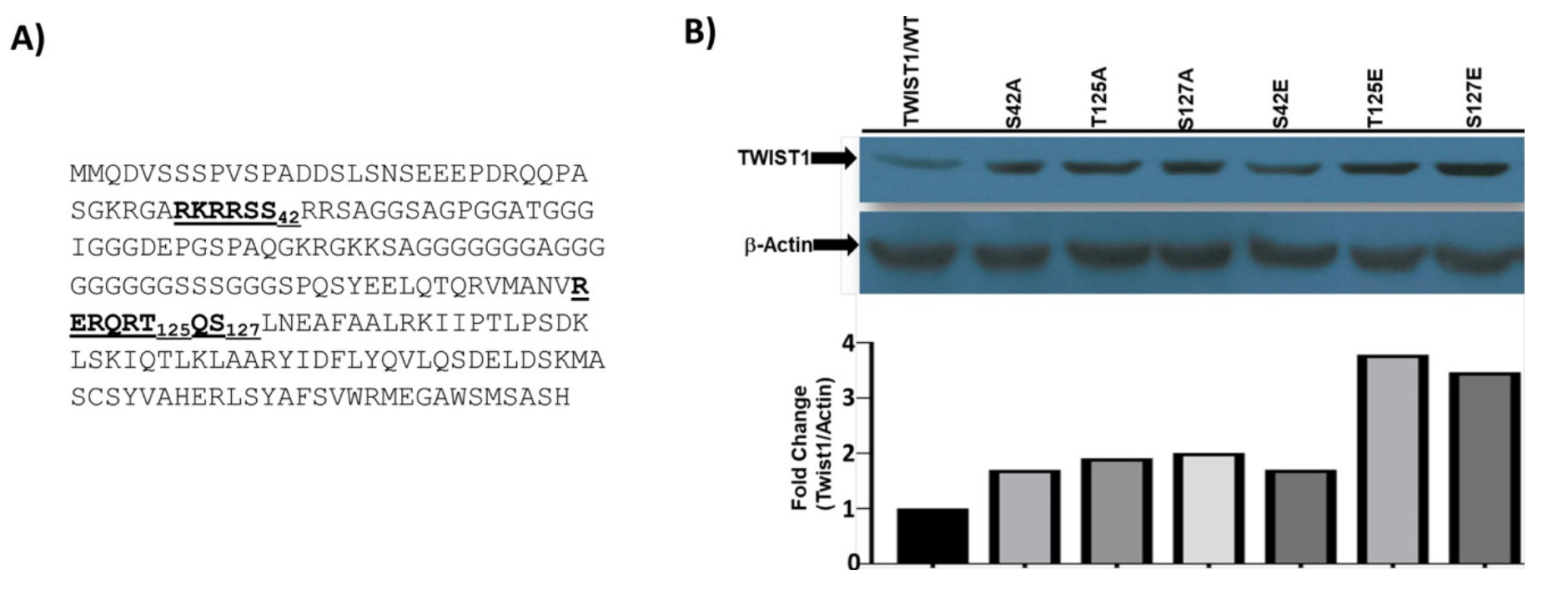
A) Amino acid sequence TWIST1 protein of *mus musculus*. AKT-phosphorylation sites (S42, T125, S127) are in bold B) Subconfluent 4T1/67NR cells were cultured DMEM in 100 mm plates were transfected with 30 ug expression vectors of alanine mutants and glutamic acid mutants of TWIST1 using calcium phosphate (Kingston et al. 2003) protocol for 72 h. Cellular lysates were collected in lysis buffer, and 100 ug total protein lysates were fractionated on %10 SDS- PAGE and blot was labeled with anti-TWIST1 antibody. The blot was stripped off and relabeled with anti-b-actin. Band intensities were determined using Image J, and relative abundance of TWIST1 level was determined by dividing TWIST1 band intensities to that of b-Actin.

Mouse TWIST1 protein has 3 Akt phosphorylation sites at S42, T125, S127 (Figure 1A). The gene has a single coding-exon; we then amplified the exon with the cloning primers and cloned as BamHI fragment into pcDNA3.1A, and sequenced for sequence confirmation, and did not find any change in the coding region. To determine whether cloned cDNA express TWIST1 proteins, the expression vector was transiently transfected into 293T cells for 48 h. Cellular lysates were prepared, 100 ug of lysates were fractionated on 10% SDS-PAGE, and immunoblot was labeled with anti-TWIST1 antibody. Since 293T cells express almost undetectable level of TWIST1, we analyzed relative expressions of our mutants with wild-type TWIST1 to determine the fold expression. As shown in Figure 1B, all TWIST1 mutants were expressed at a higher level compared to wild-type TWIST1. 

###  3.2. Phosphorylation of TWIST1 by AKT kinase is essential for TWIST1-induced metastasis. 

**Figure 2:**
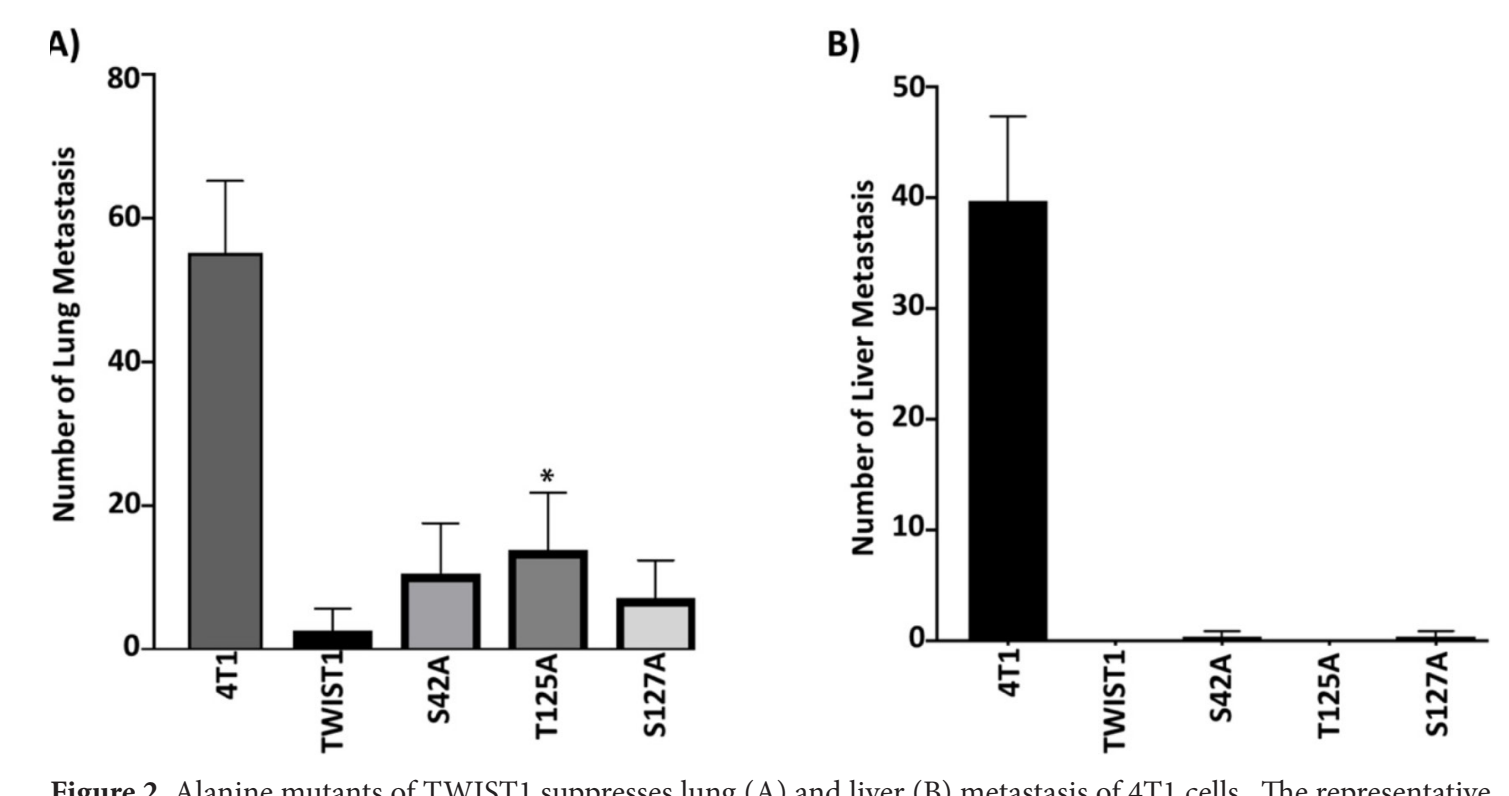
Alanine mutants of TWIST1 suppresses lung (A) and liver (B) metastasis of 4T1 cells. The representative graph shows the average number of macrometastasis in the lungs and liver. (Samples were compared with TWIST1 (WT) group for analyzing, *: P < 0.05)

**Figure 3:**
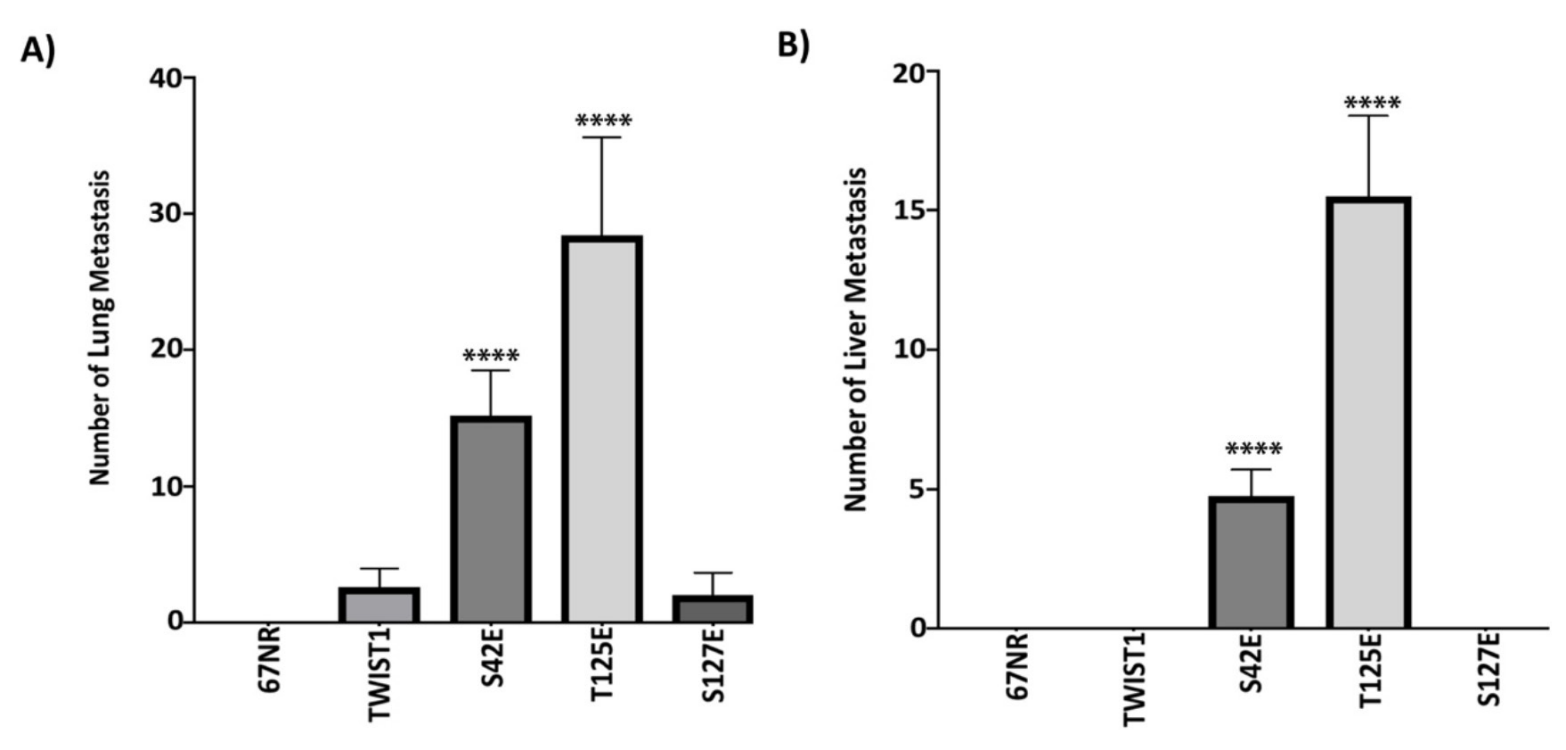
Glutamic acid mutants of TWIST1 convert nonmetastatic 67NR cells into metastatic cells. The representative graph shows the average number of macro metastasis in the lungs and liver. (Samples were compared with TWIST1 (WT) group for analyzing, ****: P < 0.0001)

We have mouse breast cancer cell lines (4T1, 67NR), and these cells share the same genetic background; however, these cells show remarkably different metastatic potential and express almost the same level of TWIST1. While 4T1 cells have very high metastatic ability to lung and liver, 67NR does not have metastatic features. According to this information, we hypothesized that alanine mutants of TWIST1 could not be phosphorylated at indicated sites, and lack of phosphorylation would interfere with the metastasis-inducing ability of TWIST1. Parallel to this, the conversation of S42, T125, and S127 to glutamic acid would create active TWIST1 and mimic the metastasis-inducing capability of TWIST1. To demonstrate this hypothesis, we stably transfected 4T1 with wild-type, and alanine mutants and 67NR cells with wild-type and glutamic acid mutants mouse TWIST1 and selected single clones for 8 weeks. Later, we injected 1 × 10^6^ 67NR and 1 × 10^5^ 4T1 cells overexpressing wild-type and mutants of TWIST1 into the breast tissue of female inbred BALB/c mice. We monitored animals for tumor growth and metastasis, at the end of the 4th week, the animals were sacrificed, lungs and livers were removed and the number of tumors larger than 1 mm was determined. As shown in Figures 2A and 2B, as expected, 4T1 cells metastasized to lung and liver with a very high number, but contrary to our expectation, ectopic expression of wild-type TWIST1 not only potentiated this, it also prevented metastasis. However, alanine mutants behaved like TWIST1 and significantly lowered lung metastasis and completely eradicated metastasis to the liver. Although the results of the ectopic expression of wild-type TWIST1 in 4T1 cells was a great surprise to us, ectopic expressions of glutamic acid mutants yielded expected results. As shown in Figures 3A and 3B, 67NR cells did not show any metastasis to lung or liver, and similar to what we observed in 4T1 cells, wild-type TWIST1 did not further induce metastasis to liver and slightly induced metastasis to lung. However, ectopic expressions of S42E and T125E mutants converted nonmetastatic 67NR to metastatic cells and caused significant metastasis to both lung and liver. 

###  3.3. Alanine mutants of TWIST1 suppress the expression of N-cadherin and vimentin in 4T1 cells. 

**Figure 4:**
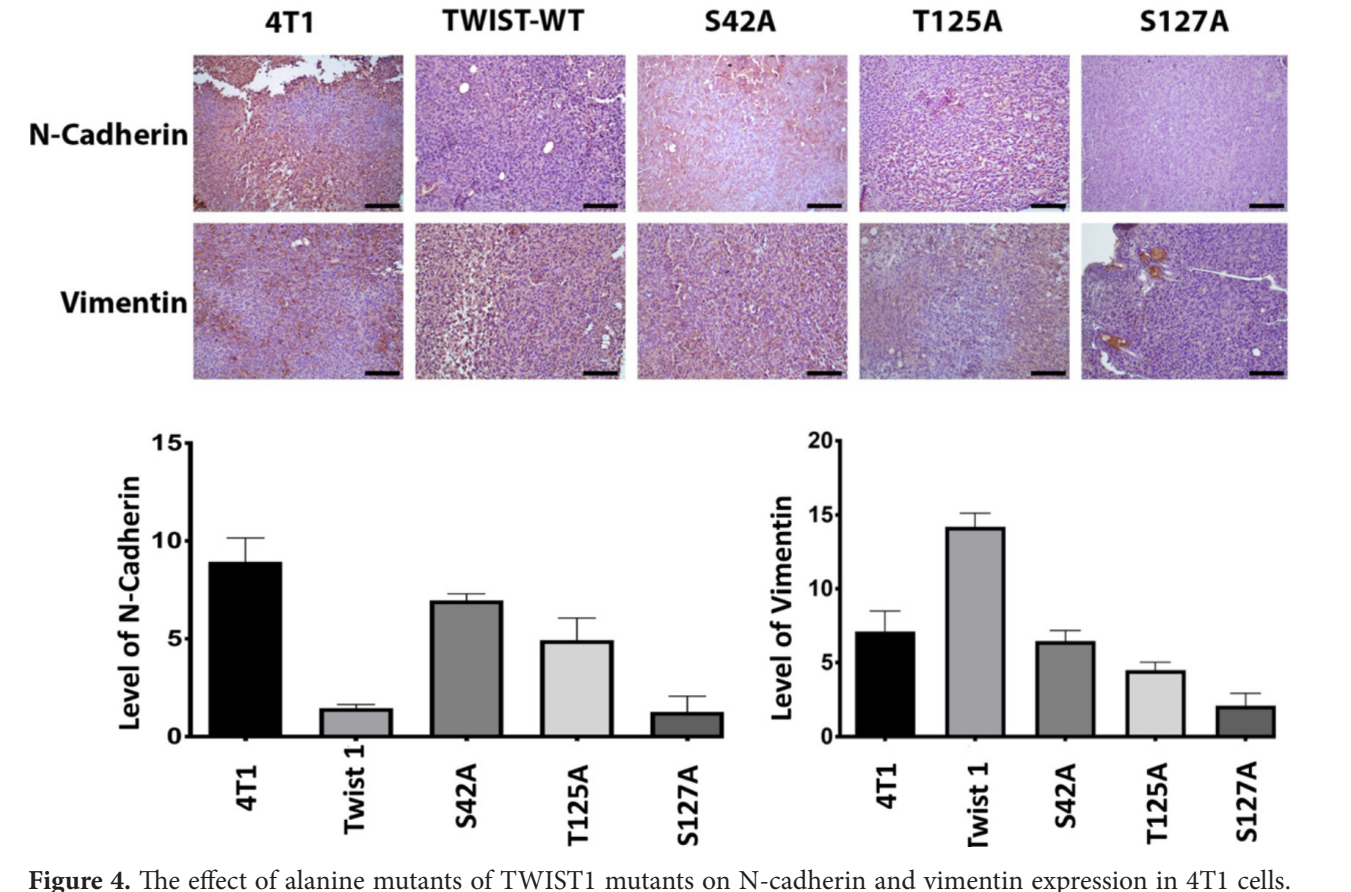
The effect of alanine mutants of TWIST1 mutants on N-cadherin and vimentin expression in 4T1 cells. 4T1 cells transfected with wild-type and alanine mutants of TWIST1 using Ca-phosphate standard procedure. Stable clones were selected with 800 μg/mL of neomycin/G418 for 8 weeks. 1 × 10^5^ 4T1 cells expressing wild-type and glutamic acid mutants of TWIST1 were injected orthotopically into the right upper mammary gland of 8–10-week- old BALB/c mice. Primary tumors were removed 27 days after injection. After the fixation of the primary tumor in 4% formaldehyde and embedded in paraffin, 5 μm thick serial sections were collected. Sections were immunostained with N-cadherin and vimentin antibody. Slides were examined using light microscopy, and analysis was done using Image J and Graphpad Prism (20× magnification) (Samples were compared with TWIST1 (WT) group for analyzing, ****: P < 0.0001)

Since we hypothesized that Akt-mediated phosphorylations of TWIST1 would affect EMT in mouse breast cancer cell lines, and alanine mutants of TWIST1 interfered with metastasis of 4T1 cells we wanted to know whether ectopic expression of alanine mutants affected the expressions of N-cadherin and vimentin. Immunohistochemical staining of tumors formed by 4T1 cells overexpressing alanine mutants showed that 4T1 cells express a significant amount of vimentin, and this was further increased by wild-type TWIST1. However, the expression of vimentin was significantly suppressed in 4T1 cells expressing alanine mutants. We saw almost the same trend in the suppression of N-cadherin expression by alanine mutants. However, TWIST1 did not further increase the expression of N-cadherin compared to 4T1 cells alone (figure 4).

### 3.4. Glutamic acid mutants of TWIST1 differentially affect the expression of N-cadherin and vimentin in 67NR cells

**Figure 5:**
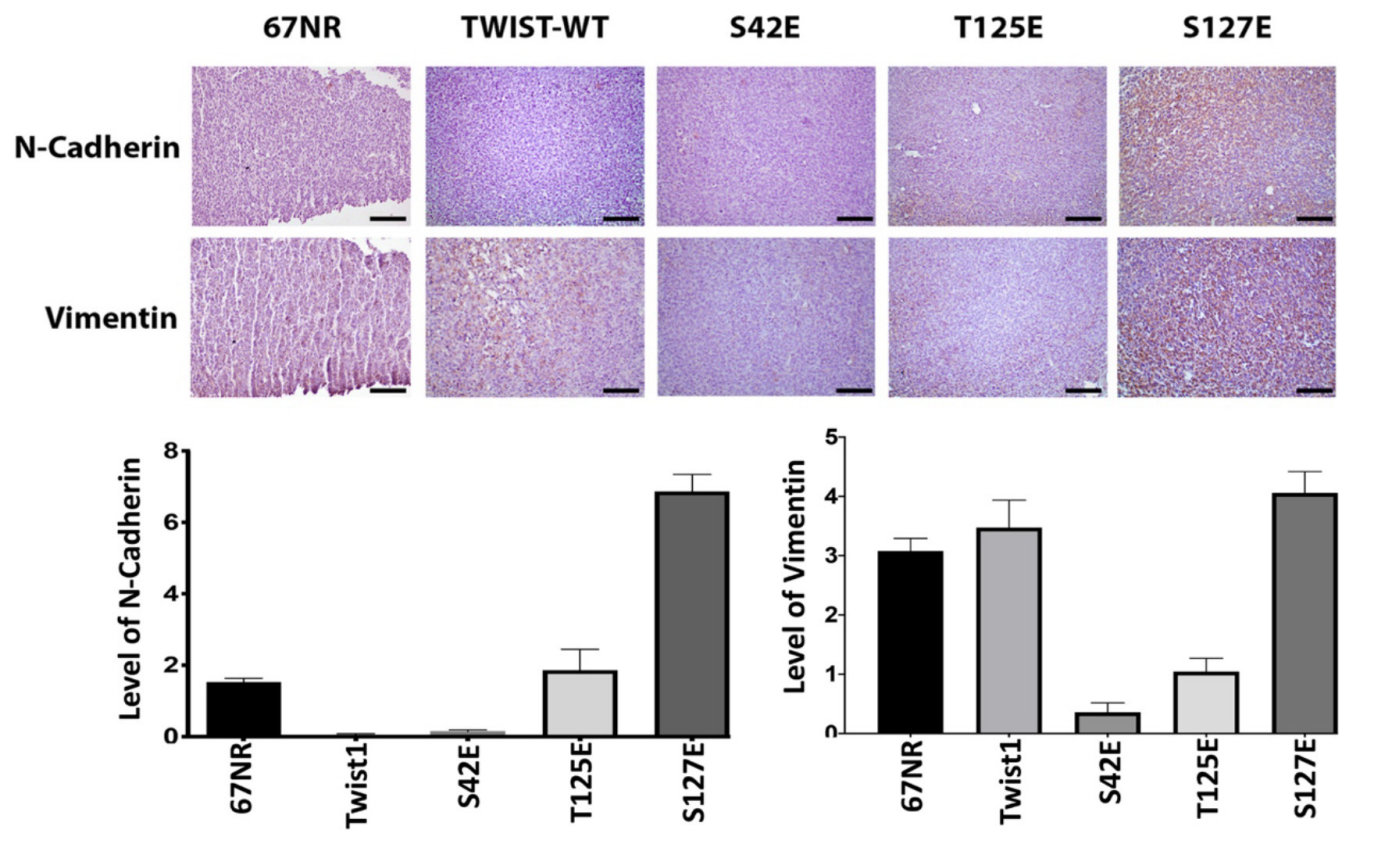
The effect of glutamic acid mutants of TWIST1 mutants on N-cadherin and vimentin expression in 67NR cells. 67NR cells transfected with wild-type and glutamic acid mutants of TWIST1 using Ca-phosphate standard procedure. Stable clones were selected with 800 μg/mL of neomycin/G418 for 8 weeks. 1 × 10^6^ 67NR cells expressing wild-type and glutamic acid mutants of TWIST1 were injected orthotopically into the right upper mammary gland of 8–10-week-old BALB/c mice. Primary tumors were removed 27 days after injection. After the fixation of the primary tumor in 4% formaldehyde and embedded in paraffin, 5 μm thick serial sections were collected. Sections were immunostained with N-cadherin and vimentin antibody. Slides were examined using light microscopy, and analysis was done using Image J and Graphpad Prism. (20× magnification) (Samples were compared with TWIST1 (WT) group for analyzing, ****: P < 0.0001)

Since there is a strong correlation between increased TWIST1 expression and EMT, and ectopic expression of S42E and T125E mutants of TWIST1 transformed nonmetastatic 67NR cells into metastatic cells, we wanted to explain the reason behind these observations. To do that, we determined the level of expression of vimentin and N-cadherin in tumor samples of 67NR cells. As shown in Figure 5, in 67NR tumors, there is a detectable level of N-cadherin and vimentin expression, and ectopic expression of wild-type TWIST1 did not significantly induce expressions of neither N-cadherin nor vimentin. In fact, contrary to our expectations, ectopic expression of wild-type TWIST1 and S42E mutants significantly lowered the expression of N-cadherin, T125E showed almost no effect. However, ectopic expression of S127E mutant induced three-fold induction of N-cadherin compared to 67NR and slightly induced vimentin expressions, although it did not induce metastasis. 

## 4. Discussion

TWIST1 is a transcription factor bearing bHLH DNA-binding domain and plays a pivotal role during embryonic development, essential for the development of somite, limb bud, and cranial neural tube in mammals. Heterozygous loss-of-function mutation (LOH) of TWIST1 causes Saethre–Chotzen syndrome both in mice and human Alexander et al. 2006 (Chen et al., 1995; Howard et al., 1997; el Ghouzzi et al*.,* 1997; Rice et al., 2000; Bialek et al., 2004; Connerney et al., 2006; Qin et al., 2012). Early studies showed that the nonredundant role of TWIST1 during embryonic bone development was explained by TWIST1-mediated inhibition of RUNX2 and MyoD1, which are responsible for osteogenic and myogenic differentiation, respectively (Funato et al., 2001; Yousfi et al., 2002; Gong et al., 2002). Although TWIST1 is essential during embryonic development, its expression is limited to fibroblasts of the mammary glands (MGs) and dermal papilla cells of the hair follicles (Xu et al., 2013). 

Previous studies showed that TWIST1 is overexpressed in the mammary glands, and its aberrant expression was correlated with chromosomal instability, in vivo angiogenesis, and metastasis of breast cancer cells (Mironchik et al., 2005; Xu et al. 2017). Recently, it was also shown that TWIST1 is phosphorylated by Akt, in turn, TWIST1 transcriptionally upregulates AKT2 gene in breast cancer cells, and this induction leads to invasion and migration of breast cancer cells (Cheng et al., 2007; Cheng et al., 2008; Xue et al., 2012). Although TWIST1 expression is limited to breast tissue and hair follicles in healthy adult, recent findings showed that overexpression and aberrant activation of TWIST1 has been linked to the development of many cancers including but not limited to gliomas, gastric cancer, hepatocellular carcinoma, prostate cancer, endometrial cancer, colon cancer, and bone cancer (Elias et al., 2005; Lee et al., 2006; Kyo et al., 2006; Alexander et al., 2006; Yan-Qi et al., 2007; Singh, 2014; Wang et al., 2018).

In line with these, we wanted to contribute to the significance of Akt-mediated phosphorylation of TWIST1 by using mouse breast cancer cell model. To directly test the impact of TWIST1 phosphorylation by Akt, we genetically modified Akt phosphorylation sites (S42, T125, S127) of TWIST1 using site-directed mutagenesis and created glutamic acid and alanine mutants of indicated sites. Since alanine mutants could not be phosphorylated, we thought that the malignant mouse breast cancer cell line (4T1) stably expressing alanine mutants of TWIST1 would lose metastatic ability when implanted into the breast tissue of BALB/c mice. Indeed, as seen in Figure 2, the ectopic expression of alanine mutants almost completely ablated metastasis of 4T1 cells to the liver and severely diminished metastasis to the lungs. Parallel to these, we thought that the nonmalignant mouse breast cancer cell line (67NR) stably expressing glutamic acid mutants of TWIST1 would gain metastatic ability when implanted into the breast tissue of BALB/c mice. As shown in Figure 3, S42E and T125E mutants showed significant metastasis to both lungs and liver. However, 67NR cells transfected with wild-type TWIST1 and S127E mutant did not show any metastasis to liver and very rare metastasis to the lungs. As anticipated, 67NR cells alone did not metastasize to either organ. 

To explain our results, we determined the levels of N-cadherin and vimentin, which are required for EMT and metastasis in tumor samples of 4T1 and 67NR cells. As shown in Figure 5, the expression levels of N-cadherin and vimentin significantly diminished in 4T1 cells expressing alanine mutants, and these results can explain as to why 4T1 cells expressing alanine mutants cannot metastasize. However, S42E and T125E expressing 67NR cells did not show a significant increase in N-cadherin and vimentin expressions, although these cells showed significant metastasis to lungs and liver.

 Taken together, our results show that Akt-mediated phosphorylation of TWIST1 can revert the nonmetastatic phenotype of breast cancer cells into metastatic phenotype and changes in expression of vimentin and N-cadherin may not be enough to explain EMT. Since TWIST1 expression is limited to specific organs in adults, it is conceivable that TWIST1 would be a cancer-preferential drug target to prevent metastasis of breast cancer. 

Supplementary Materials
