# Supplementary material for: AKT-mediated phosphorylation of TWIST1 is essential for breast cancer cell metastasis
Source: Turk J Biol. 2020 Aug 19;44(4):158–65. doi: 10.3906/biy-1912-74 (PMC7478131; doi:10.3906/biy-1912-74)
Supplement: Supplementary file 1 — Supplementary Materials [file turkjbio-44-158-sup001.pdf]

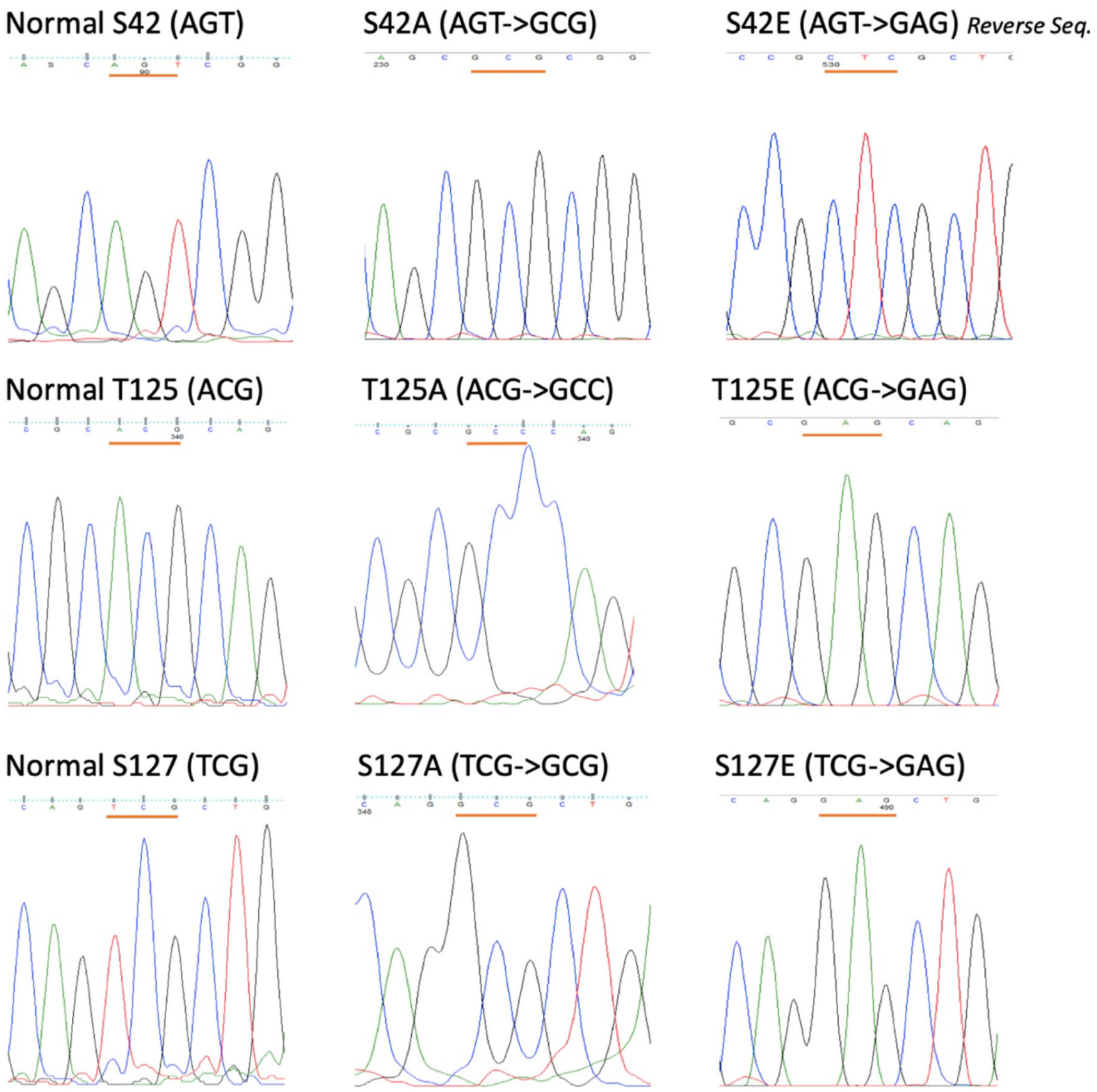

Figure S1. Confirmation of mutations obtained by Site-Directed Mutagenesis with sanger sequence.
